# Can proportional ventilation modes facilitate exercise in critically ill patients? A physiological cross-over study: Pressure support versus proportional ventilation during lower limb exercise in ventilated critically ill patients

**DOI:** 10.1186/s13613-017-0289-y

**Published:** 2017-06-12

**Authors:** Evangelia Akoumianaki, Nicolas Dousse, Aissam Lyazidi, Jean-Claude Lefebvre, Severine Graf, Ricardo Luiz Cordioli, Nathalie Rey, Jean-Christophe Marie Richard, Laurent Brochard

**Affiliations:** 1 Intensive Care Unit, University Hospital of Heraklion, Heraklion, Crete, Greece; 2 Division of Intensive Care, Geneva University Hospitals, Geneva, Switzerland; 3 Institut Supérieur des Sciences de la Santé, Laboratory Rayonnement-Matiére et Instrumentation, Université Hassan 1er, Settat, Morocco; 4 Department of Anesthesiology and Critical Care, Université Laval, Quebec, QC Canada; 5 Department of Adult Intensive Care Unit, Hospital Israelita Albert Einstein, São Paulo, Brazil; 6 Department of Anesthesia and Intensive Care Unit, Rouen University Hospital, Rouen, France; 7 Emergency Department, General Hospital of Annecy, Annecy, France; 8 INSERM UMR 955, Créteil, France; 9 Keenan Research Centre, Li Ka Shing Knowledge Institute, St. Michael’s Hospital, Toronto, Canada; 10 Interdepartmental Division of Critical Care Medicine, University of Toronto, Toronto, ON Canada

**Keywords:** Critically ill patients, Exercise, Assisted mechanical ventilation, Proportional ventilation, Oxygen consumption, Work efficiency

## Abstract

**Background:**

Early exercise of critically ill patients may have beneficial effects on muscle strength, mass and systemic inflammation. During pressure support ventilation (PSV), a mismatch between demand and assist could increase work of breathing and limit exercise. A better exercise tolerance is possible with a proportional mode of ventilation (Proportional Assist Ventilation, PAV+ and Neurally Adjusted Ventilatory Assist, NAVA). We examined whether, in critically ill patients, PSV and proportional ventilation have different effects on respiratory muscles unloading and work efficiency during exercise.

**Methods:**

Prospective pilot randomized cross-over study performed in a medico-surgical ICU. Patients requiring mechanical ventilation >48 h were enrolled. At initiation, the patients underwent an incremental workload test on a cycloergometer to determine the maximum level capacity. The next day, 2 15-min exercise, at 60% of the maximum capacity, were performed while patients were randomly ventilated with PSV and PAV+ or NAVA. The change in oxygen consumption (ΔVO_2_, indirect calorimetry) and the work efficiency (ratio of ΔVO_2_ per mean power) were computed.

**Results:**

Ten patients were examined, 6 ventilated with PSV/PAV+ and 4 with PSV/NAVA. Despite the same mean inspiratory pressure at baseline between the modes, baseline VO_2_ (median, IQR) was higher during proportional ventilation (301 ml/min, 270–342) compared to PSV (249 ml/min, 206–353). Exercise with PSV was associated with a significant increase in VO_2_ (ΔVO_2_, median, IQR) (77.6 ml/min, 59.9–96.5), while VO_2_ did not significantly change during exercise with proportional modes (46.3 ml/min, 5.7–63.7, *p* < 0.05). As a result, exercise with proportional modes was associated with a better work efficiency than with PSV. The ventilator modes did not affect patient’s dyspnea, limb fatigue, distance, hemodynamics and breathing pattern.

**Conclusions:**

Proportional ventilation during exercise results in higher work efficiency and less increase in VO_2_ compared to ventilation with PSV. These preliminary findings suggest that proportional ventilation could enhance the training effect and facilitate rehabilitation.

**Electronic supplementary material:**

The online version of this article (doi:10.1186/s13613-017-0289-y) contains supplementary material, which is available to authorized users.

## Background

Mechanical ventilation (MV) represents one of the most common therapeutic strategies in critically ill patients [1, 2]. Mechanically ventilated patients are traditionally considered too sick for early physical therapy and mobilization. It has been demonstrated that a loss of muscle mass and strength rapidly occur from the first days of bed rest together with insulin resistance and inflammatory process [3–5]. Therefore, the high rates of neuromuscular weakness encountered in mechanically ventilated patients result not only from the illness itself, but also from the immobility associated with mechanical ventilation [3, 6, 7]. ICU-acquired weakness has been correlated with worse acute morbidity, increases healthcare-related costs and increased mortality [8–10]. It has been shown that, early exercise of critically ill patients is both safe and beneficial on muscle strength, muscular mass preservation and on systemic inflammatory suppression [11–14]. Not all studies have shown positive effects [15], and it may be important to start rehabilitation very early, even at a stage of passive mobilization.

The type of ventilatory mode may significantly affect exercise performance. Inefficient mechanical unloading and patient-ventilator asynchronies may limit the tolerance to exercise in various ways, including through work of breathing and perceived dyspnea [16]. A better exercise tolerance could be expected with a proportional mode of ventilation, which can translate into longer and more efficient training sessions.

In pressure support ventilation (PSV), the ventilator assistance remains fixed for every breath and does not adjust to changing ventilator demand. A mismatch between demand and assist could promote patient-ventilator asynchrony, increase work of breathing and eventually limit exercise [17]. Proportional ventilator modes (Proportional Assist Ventilation with load-adjustable gain factors, PAV+ and Neurally Adjusted Ventilatory Assist, NAVA) are theoretically advantageous in that patient inspiratory effort drives the assistance provided by the ventilator proportionally [18]. NAVA [19, 20] and PAV+ [21, 22] have been shown to preserve normal breathing pattern, to reduce asynchronies and to better unload inspiratory muscles in the presence of varying ventilator demand compared with PSV [23]. Exercising with noninvasive PAV allowed greater endurance time in various populations [24–27].

In this prospective randomized preliminary blinded study, we aimed to examine whether, in critically ill patients, PSV and proportional ventilation modes have different effects on respiratory muscle unloading and work efficiency during exercise. Secondarily, we explored potential differences on breathing pattern, patient comfort and patient-ventilator synchrony. Because such studies are difficult to perform, we did not plan to specifically compare the two proportional modes but simply to see whether the two offered the same feasibility.

## Methods (see also Additional file 1: Supplemental Digital Content, SDC)

### Patients

The study took place at the Geneva university hospital ICU, a mixed medico-surgical ICU. Over an 18-month period (December 2011–May 2013), critically ill patients requiring assisted MV for more than 48 h were prospectively enrolled. The study was approved by the hospital ethics committee (protocol n 11-224, accepted 05.12.11), and informed consent was obtained from the patients or their families.

### Study protocol

As study day 1 was defined the day of study initiation. All patients had been already ventilated for variable durations before study day 1 as shown in Table 1. At study day 1, the patients underwent an incremental workload test on a cycloergometer to determine the maximum level capacity. This started from passive mobilization on the bicycle, and the resistance was then gradually increased based on patient’s tolerance. During this test, patients were ventilated with PSV. The next day (study day 2), two 15-min exercise periods, at 60% of the maximum resistance, were performed. During each session, patients were ventilated with PSV and a proportional mode in a random order. The type of proportional mode (PAV+ or NAVA) was randomly selected. The physiotherapist supervising the exercise test was blinded to the attributed sequence. Only the investigator in charge of collecting the data was aware of the mode. The exercise period was terminated prematurely if one of the predefined stopping criteria was met (see SDC).

**Table 1:** Patient characteristics

Patient	Sex	Age (years)	Diagnosis	ICU (days)	MV (days)	APACHE II	PS	PEEP	FiO_2_ (%)	Group
1	M	69	Sepsis-MOF	14	14	29	4	6	24	NAVA
2	M	53	Cardiac arrest	54	54	42	7	5	30	NAVA
3	F	53	CO intoxication	6	6	16	13	5	35	NAVA
4	M	66	Pneumonia	14	14	20	5	5	30	NAVA
5	M	54	Esophageal cancer	7	2	25	11	6	27	PAV+
6	M	72	Polytrauma	19	19	13	9	8	28	PAV+
7	M	61	ARF	32	32	22	16	7	21	PAV+
8	F	55	AECOPD	16	16	28	8	5	34	PAV+
9	F	55	Sepsis–cirrhosis	12	12	13	9	5	30	PAV+
10	M	48	Pneumonia	14	14	18	7	6	35	PAV+
Median		55		14	14	21				
IQR		53–65		12.5–18.3	12.5–18.3	16.5–27.3				

*M* male, *F* female, *ARF* acute respiratory failure, *MOF* multiple organ failure, *CO* carbon monoxide, *AECOPD* acute exacerbation of chronic obstructive pulmonary disease, *ICU* intensive care unit, *MV (days)* duration of mechanical ventilation (in days) at first study day, *APACHE II* simplified acute physiology score II, *PS* ventilator assistance during baseline ventilation, *PEEP* positive end expiratory pressure, *PAV+* Proportional Assist Ventilation with load-adjustable gain factors, *NAVA* neurally adjusted ventilator assist, *IQR* interquartile range

Ventilator settings during the exercise sessions are described in the Additional file 1: Supplemental Digital Content. The ventilator support was titrated to attain the same mean airway pressure (Paw_mean_) in all modes tested [28]. Most patients had a tracheostomy at the time of the study. The size of the endotracheal or tracheostomy tube did not differ between the modes tested, and its internal diameter was entered to the ventilator before study commencement.

### Equipment

**Fig. 1:**
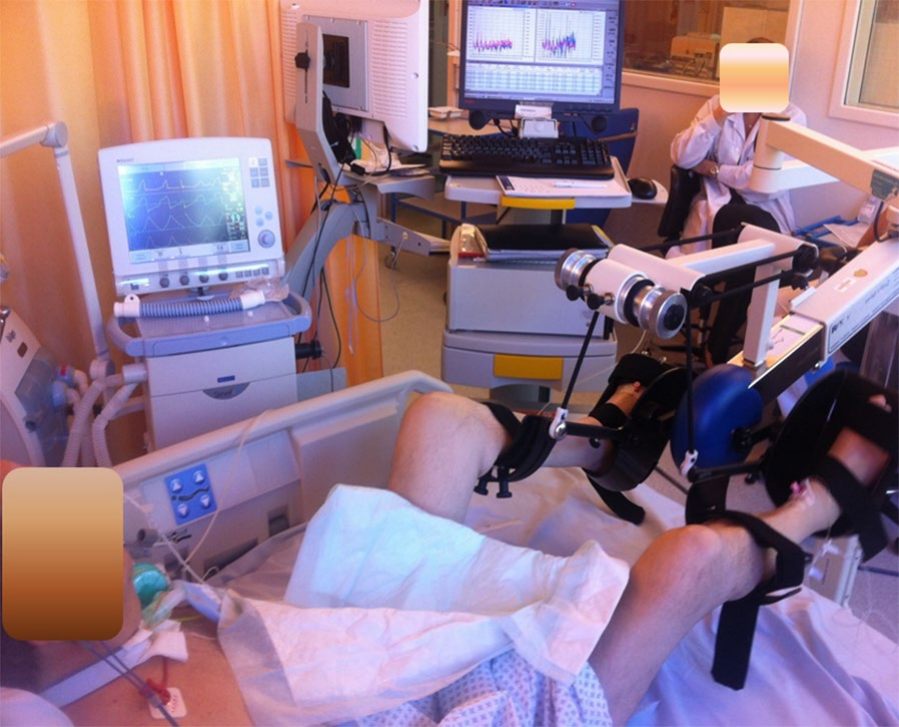
Example of a patient ventilated with Neurally Adjusted Ventilator Assist (NAVA) while performing exercise with the cycle ergometer (MOTOmed Letto 2, RECK-Technik GmbH and Co. Betzenweiler, Germany). Oxygen consumption is measured through indirect calorimetry

All exercise tests were made using the same bedside cycle ergometer (Fig. 1) (MOTOmed Letto 2, RECK-Technik GmbH and Co. Betzenweiler, Germany). Patients randomized to PSV/PAV+ were ventilated with a PB 840 ventilator (Covidien, Mansfield, MA, USA). Patients randomized to PSV/NAVA were ventilated with a Servo-i ventilator (Maquet, Solna, Sweden). An indirect calorimetry apparatus was used to measure oxygen uptake (Quark RMR ICU; Cosmed, Rome, Italy).

Airway flow and pressure sensors were connected to the respiratory circuit, proximal to the Y-piece. The flow was measured through a pneumotachograph (Fleish No. 2; Metabo; Epalinges, Switzerland). Proximal airway pressure was measured using a differential pressure transducer (Validyne MP45 ±80cmH_2_O; Northridge, CA, USA). Signals were acquired with an analogue–digital converter (MP100; Biopac systems, Goleta, CA, USA), sampled at 200 Hz and stored in a laptop computer for subsequent off-line analysis (Acqknowledge 3.7.3, Biopac Systems).

### Data collection and study end points

Exercise performance expressed as mean (Wmean) and peak (Wpeak) power generated during exercise (in watts), distance performed (km) and exercise time (min) were manually collected from the cycle ergometer. Dyspnea and limb fatigue were evaluated through a modified Borg dyspnea scale score and a Borg limb discomfort scale. Oxygen consumption (VO_2_) and CO_2_ production (VCO_2_) were measured through indirect calorimetry. Work efficiency, our primary end point, was indicated by the ratio of the change of VO_2_, in absolute values, to the mean power generated (Wmean, in watts) during the exercise session [29]. Higher work efficiency is indicated by a lower ΔVO_2_/W ratio. Respiratory [respiratory rate (RR) and SpO_2_)] and hemodynamic parameters [heart rate (HR), arterial blood pressure and double product (SBD * HR)] were continuously monitored. Breath-by-breath analysis was performed on the recorded data by using Acqknowledge^®^ software (Biopac Systems Inc., Goleta, CA, USA). To assess the severity of asynchrony, we used the asynchrony index (AI) [17] (see SDC).

### Statistical analysis

Due to the small sample size, we used three approaches to mitigate the risk of false discovery: (1) We used nonparametric tests which are less sensitive and less powerful than parametric tests. Continuous variables were expressed as medians (25–75th interquartile range, IQR); (2) we started all comparisons by performing a global nonparametric analysis of variance and used post hoc tests with appropriate corrections only when the analysis of variance allowed it. Variables were compared using the Friedman test for repeated measurements followed, when indicated, by a pairwise comparison with Wilcoxon signed rank using Bonferroni post hoc correction; (3) we did a sensitivity analysis excluding one potential out-layer patient to show that the results were robust.

Statistical tests were two sided, and a *p* < 0.05 was considered statistically significant.

## Results

### Patient characteristics

**Fig. 2:**
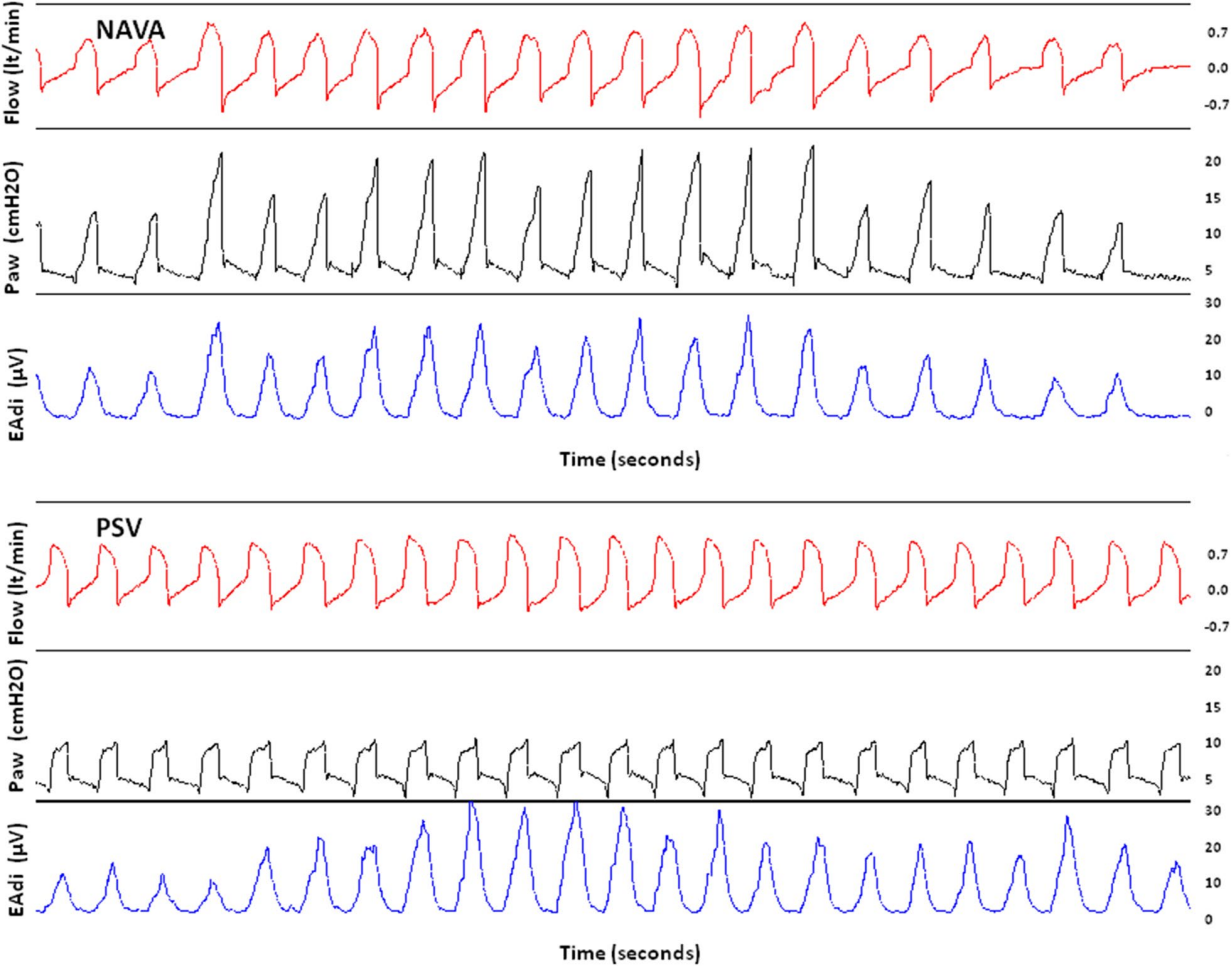
Recordings of Flow, airway pressure (Paw) and electrical activity of the diaphragm (EAdi) during exercise in the same patient during two examined sessions: one with Neurally Adjusted Ventilator Assist (NAVA) and one with pressure support ventilation (PSV). During NAVA, one can observe the great variability of Flow due to changes in neural effort (reflected by changes in EAdi) which led to changes in delivered Paw. In contrast, despite similar changes in EAdi during PSV, delivered Paw and Flow remained stable

Ten patients were enrolled; four were randomized in the group of NAVA/PSV and six in the group of PAV+/PSV. All patients completed the study. The main patient characteristics are summarized in Table 1. For the sake of clarity, we present proportional mode examined as NAVA in patients numbered 1–4 and PAV+ in patients numbered 6–10. Representative recordings of Paw and Flow with PSV and NAVA are illustrated in Fig. 2.

There was no significant difference (*p* 0.65) in the mean inspiratory Paw between PSV and proportional modes indicating adequate titration of the assistance provided (Table 2).

**Table 2:** Respiratory pattern and hemodynamic parameters

Variable tested	Baseline (proportional)	Exercise (proportional)	Baseline (PSV)	Exercise (PSV)
VO_2_ (ml/min)	301 (270–342)	335 (332–377)	249 (206–353)^§^	337 (291–402)^#^
Mean Watt	NA	2 (1, 2)	NA	1.5 (1, 2)
Mean Paw (cm H_2_O)	11 (9–14)	11 (9–15)	12 (10–14)	12 (10–14)
*V*_T_ (ml)	434 (342–581)	503 (380–581)	430 (300–491)	442 (314–638)
RR (br/min)	28 (19–34)	27 (22–35)	29 (21–31)	30 (24–37)
*V*_E_ (L/min)	9.9 (9.1–11.9)	11.3 (10.3–15.5)^#^	9.6 (8.7–11.7)	11.2 (10.0–15.0)^#^
SBP (mmHg)	117 (106–140)	119 (112–147)	122 (101–141)	125 (117–156)
DBP (mmHg)	68 (55–78)	66 (53–79)	68 (53–83)	71 (55–82)
HR (bpm)	102 (74–114)	104 (77–118)	100 (78–109)	108 (83–118)^#^
HR * SBP (bpm * mmHg)	11,385 (9344–13,309)	12,053 (10,045–14,567)^#^	11,400 (9320–13,229)	13,772 (10,148–16,220)^#^
SpO_2_ (%)	98 (96–100)	96 (93–100)	97 (96–99)	96 (93–99)

Data are median (interquartile range). *VO*_*2*_ oxygen consumption, *NA* not applicable, *V*_*T*_ tidal volume, *RR* patient respiratory rate, *V*_*E*_ minute ventilation, *SBP* systolic blood pressure, *DBP* diastolic blood pressure, *HR* heart rate, *SpO*_*2*_ oxygen saturation, *PSV* pressure support ventilation

^#^ *p* < 0.05, baseline versus exercise

^§^ *p* < 0.05, PSV versus proportional modes

### Primary outcome: oxygen consumption and work efficiency

**Fig. 3:**
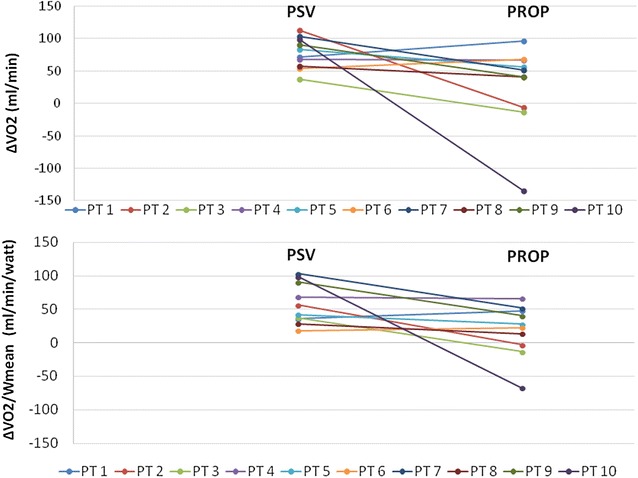
Changes in oxygen consumption (ΔVO_2_) and ΔVO_2_/Wmean before and after exercise between the modes tested. ΔVO_2_; difference in VO_2_ between the start and the end of exercise in absolute values. *PROP* proportional ventilation, *PSV* pressure support ventilation

VO_2_ and Watt are presented in Table 2. At baseline, VO_2_ was significantly lower between PSV and proportional modes, while the VO_2_ at exercise did not differ between the compared ventilator modes. Oxygen consumption increased significantly during exercise with PSV (∆VO_2_ median, IQR 77.6 ml/min, 59.9–96.5), while it did not change during exercise with proportional modes (46.3 ml/min, 5.7–63.7, *p* < 0.05, Fig. 3). ΔVO_2_/W (median, IQR) during exercise with PSV was, on average, almost two times higher than the value recorded with proportional modes (49.2 ml/min/W, 36.2–85.2, vs. 25.4 ml/min/W, 1.2–46.1, *p* < 0.05). After excluding patient 10 (the patient exhibiting the highest VO_2_ decrease at the end of exercise with PAV) from the analysis, the difference in the main outcome (ΔVO_2_/W) remained statistically significant between proportional modes and Pressure Support (*p* = 0.038). There was a statistically significant increase in VCO_2_ during ventilation with PSV (from 194.2 ml/min, 175.1–285.0 before exercise to 258.7 ml/min, 223.5–338.2 at the end of exercise). VCO_2_ (median, IQR) did not significantly change between the beginning (230.4 ml/min, 216.6–291.8) and the end of exercise (282.7 ml/min, 254.6–316.3) during proportional ventilation.

### Secondary outcomes

Exercise significantly increased minute ventilation (VE) with no significant effect of the ventilator mode on respiratory pattern and SpO_2_ (Table 2). Four patients had a baseline RR of ≥30 breaths/min both at PSV and proportional ventilation. The patients exhibited no signs of respiratory distress, and the breathing frequency remained unaffected by ventilator assistance modification.

There was an increase in HR and double product following exercise (Table 2). Hemodynamic parameters were not influenced by the ventilatory mode. Half of the patients had no major asynchrony events. The highest AI observed was 9%. Median (IQR) AI in all patients was 0% (0–3.5%) and did not differ between the modes tested.

The ventilator mode had no significant effect on patient’s perception of dyspnea, limb fatigue or the final distance cycled (Table 1 SDC).

## Discussion

To our knowledge, this is the first study comparing, in critically ill patients, PSV to proportional ventilation during exercise. Despite a difference in baseline VO_2_, we found that patients ventilated with PSV exhibited a significant increase in VO_2_ during exercise while VO_2_ did not change compared to baseline value during exercise with proportional ventilation. As a result, proportional ventilation was associated with a better work efficiency (ΔVO_2_/Wmean) than PSV. The ventilation mode did not affect the breathing pattern, the patient’s perception of dyspnea and limb fatigue, the hemodynamic parameters or patient-ventilator asynchrony.

There is evidence that critical illness and associated bed rest induce muscular weakness through various interrelated pathophysiological mechanisms, mainly the release of reactive oxygen species and inflammatory cytokines, which promote muscle catabolism and reduce muscle protein synthesis. The resulting muscle loss and weakness prolong mechanical ventilation and ICU and hospital stay and decrease patient’s functional status and quality of life, even years following hospital discharge [30]. Early intervention with exercise in the ICU is feasible, safe, prevents muscle mass loss and decreases oxidative stress and inflammation [31]. With respect to clinical outcomes, it is associated with shorter duration of mechanical ventilation, a decrease in the ICU and hospital length of stay and improved quality of life [11, 12, 14, 32–34]. On the contrary, if patient mobilization is not undertaken early in the ICU, hospital readmission and rates of death during the first year following hospital discharge increase [35]. One recent ICU study did not show improvement with a rehabilitation program, but it is possible that it was not started early enough [15].

Despite recommendations [36], clinicians remain reluctant to implement early mobilization. Only 12.5–27% of patients with acute respiratory failure receive any physical therapy in the ICU [12, 37]. The patient’s respiratory status can be a limiting factor, interfering with exercise performance and potentially with rehabilitation [16, 38]. Patients during their weaning phase are mostly ventilated in PSV, in which the assistance provided remains fixed for every breath cycle. This could be problematic during exercise, because patient’s demand varies dynamically over a short period of time. By promptly adapting their assistance to patient changing ventilator demands, proportional modes like PAV+ and NAVA can be advantageous in this setting. PAV has been shown to increase endurance capacity and improve exercise tolerance in stable COPD patients [24, 27]. However, there was no evidence on the influence of ventilator mode in exercise capacity during the ICU stay. This study is the first to show a difference in favor of proportional modes during exercise in critically ill patients. Ventilation with PSV was associated with a significant increase in VO_2_ during exercise, while there was no difference in VO_2_ between baseline and exercise with proportional ventilation. As a result, proportional ventilation was associated with a better work efficiency (ΔVO_2_/Wmean) compared to ventilation with PSV. Less increase in VO_2_ can be interpreted as a lower O_2_ consumption by the respiratory muscles and, hence, more efficient unloading of patient’s work of breathing. On a long-term basis, this could allow patients to perform more exercise with proportional ventilation than with traditional modes.

There were no significant changes in respiratory rate, blood pressure and SpO_2_ between periods of rest and exercise. Exercise slightly increased heart rate and double product. Our results are in agreement with previous reports [11, 39] as well as the results of a more recent case series which examined the physiological aspects and safety of early passive cycling exercise in mechanically ventilated patients [40]. The stability of the hemodynamic and respiratory pattern variables further supports the safety of mobilization of the mechanically ventilated critically ill patients.

For safety reasons, we aimed at an exercise reproducing only 60% of the maximum resistance level attained the day before the sessions and a duration of only 15 min. Therefore, the significant improvement in work efficiency with proportional modes was not translated to a reduction in patient’s perception of dyspnea, limb fatigue or to a higher distance performed. During physical exercise, a competition for blood flow takes place between the diaphragm and the locomotor muscles [41, 42]. A respiratory muscle load-induced metaboreflex has been described, which increases the sympathetic tone and reduces the perfusion toward the limb muscles [16]. Decreasing the work of breathing with MV has been linked with increased blood flow in the limbs and less locomotor muscle fatigue [43]. To the contrary, experimentally increasing the work of breathing increased severity of quadriceps fatigue [24, 43]. Diaphragmatic fatigue was associated with increased sympathetic activity in the legs and consequential reduction of blood flow [43]. While these negative effects were found during heavy sustained exercise, the results have been less consistent at submaximal exercise, probably because fatiguing contractions are needed to provoke the metaboreflex [44]. Furthermore, it has been demonstrated that dyspnea intensity is not influenced by VO_2_ requirements, while it is strongly correlated with respiratory neural drive and increases as a function of increasing VCO_2_ [45, 46].

Patient-ventilator asynchronies were rare in the population studied. None of the patients studied had an AI of 10% or more. With such a low rate of asynchronies, a superiority of one ventilator mode with regard to patient-ventilator interaction would not be apparent. The low rate of asynchronies can be explained by the small patient population and the relative short study period. The incidence of ineffective efforts varies over time in the same patient [47]. In addition, it has been observed that asynchronies tend to occur in clusters, between often prolonged uneventful periods [48]. Finally, only one patient had COPD while the median VT in studied modes was low. COPD and high levels of pressure support and VT are factors that further increase the incidence of major asynchronies, notably ineffective efforts [49].

### Methodological comments and study limitations

Despite targeting a breathing frequency under 30 breaths/min, as described in the methodology, four patients had a baseline RR of ≥30 breaths/min both at PSV and proportional ventilation. The patients exhibited no signs of respiratory distress, and the breathing frequency remained unaffected by ventilator assistance modification. The RR observed may have been the unstressed value preferred by the patient’s control system [28].

Similar to most studies comparing ventilator modes, we titrated the ventilator assistance to target the same mean Paw between proportional and PSV ventilation at baseline [22, 50, 51]. Apart from Paw_mean_, baseline VT and VE were also similar between the modes tested. Although targeting the same mean inspiratory Paw (close to the peak Paw) is a classical way to titrate the assistance in NAVA, the baseline VO_2_ was lower during PSV compared to NAVA, suggesting that the same mean Paw may lead to a lower level of unloading during NAVA compared to PSV. This finding was unexpected based on the published literature at the time of the study. However, it is in accordance with the data published by Carteaux et al., who clearly showed that, for the same peak Paw or even for a peak Paw 20% higher in NAVA than in PSV (i.e., approximately the same mean Paw), the unloading was less with NAVA than with PSV [52]. Moreover, a more recent study from our group performed in an active lung model demonstrated that PAV+ delivered a lower mean Paw from the mean Paw which should delivered based on the equation of motion [53]. This leads to a 25% less unloading compared to its theoretical level of unloading. This was a preliminary study, and the aforementioned data were unavailable during its design. The higher baseline VO_2_ during proportional modes represents a drawback of the current study. Nevertheless, we do not think it invalidates our results but, in a sense, it limits its generalizability. In another sense, it corresponds to how clinicians usually set these proportional modes.

We chose to compare PSV with either NAVA or PAV+ instead of choosing only one proportional mode, because we aimed to explore potential differences of proportional versus pressure support ventilation on exercise performance. Since NAVA and PAV+ both resemble proportional ventilation, choosing one of them would only limit our experience to one single mode and one single brand. This would be an issue regarding the difficulty to recruit such patients. Additionally, our primary end point was the effect of ventilator mode on work efficiency and not peak VO_2_. A number of factors such as circulatory impairment, muscle fiber type, body mass index, breathing reserve and peak heart rate may strongly influence the linearity between VO_2_ and work relationship [54, 55]. Hence, it has been proposed to use work efficiency as a better reflection of exercise tolerance [54, 56, 57]. We used the cycle ergometer, as already in use in our ICU to mobilize critically ill patients, and it offers the advantage to strictly control and monitor the workload performed by the patient [11, 58].

This was a single center study, and the population studied was small suggesting that results should be interpreted cautiously. As a result of the small patient number, we have chosen nonparametric tests to analyze our results. However, our patients’ characteristics reflect the critically ill patient commonly encountered in the ICU setting. We took important precautions to ensure a reliable interpretation such as randomizing all sequences and blinding the physiotherapist and the patient. Furthermore, when performing the off-line analysis of oxygen consumption and work efficiency, the investigator was not aware of the session analyzed. Since one of our patients (Patient 10) exhibited a striking decrease in VO_2_ at the end of exercise with proportional ventilation, we reanalyzed our results excluding this patient to preclude an outlier effect. Work efficiency remained statistically significant lower (*p* = 0.038) with proportional ventilation in the remaining 9 patients tested. In addition, our results during PSV are exactly as expected, reinforcing the validity of our results. Therefore, it is likely that the results are real as the difference in work efficiency between proportional modes and PSV was significant, implying a strong effect of ventilation on exercise capacity. Finally, the small study size did not allow us to compare PAV+ with NAVA during exercise except to show a similar feasibility between the two.

## Conclusions

In a small group of critically ill patients, ventilation with PSV was associated with a significant increase in oxygen consumption while oxygen consumption did not change between rest and exercise during proportional ventilation. This resulted in less work efficiency during PSV. The findings of this preliminary study suggest that, in mechanically ventilated patients, proportional ventilation could enhance the training effect and facilitate rehabilitation in comparison with ventilation with PSV.

## Additional file

**Additional file 1: Table S1.** Results on dyspnea, limb fatigue and distance cycled. Data are presented as median (IQR) values. PROP; Proportional ventilator mode (either Proportional Assist Ventilation or Neurally Adjusted Ventilator Assist). PSV; Pressure Support Ventilation.

## Abbreviations

MV: mechanical ventilation
PSV: pressure support ventilation
PAV+: Proportional Assist Ventilation with load-adjustable gain factors
NAVA: Neurally Adjusted Ventilatory Assist
Paw_mean_: mean airway pressure
Wmean: mean power generated during exercise
Wpeak: peak power generated during exercise
VO_2_: oxygen consumption
VCO_2_: CO_2_ production
∆VO_2_: absolute increase in oxygen consumption
ΔVO_2_/W: work efficiency
RR: respiratory rate
HR: heart rate
AI: asynchrony index
VE: minute ventilation

## References

[1] Carson SS, Bach PB, Brzozowski L, Leff A (1999). Outcomes after long-term acute care: an analysis of 133 mechanically ventilated patients. Am J Respir Crit Care Med.

[2] Wunsch H, Linde-Zwirble WT, Angus DC, Hartman ME, Milbrandt EB, Kahn JM (2010). The epidemiology of mechanical ventilation use in the United States. Crit Care Med..

[3] Kortebein P, Ferrando A, Lombeida J, Wolfe R, Evans WJ (2007). Effect of 10 days of bed rest on skeletal muscle in healthy older adults. JAMA.

[4] Truong AD, Fan E, Brower RG, Needham DM (2009). Bench-to-bedside review: Mobilizing patients in the intensive care unit—from pathophysiology to clinical trials. Crit Care.

[5] Bloomfield SA (1997). Changes in musculoskeletal structure and function with prolonged bed rest. Med Sci Sports Exerc.

[6] Goldhill DR, Badacsonyi A, Goldhill AA, Waldmann C (2008). A prospective observational study of ICU patient position and frequency of turning. Anaesthesia.

[7] Ferrando AA, Lane HW, Stuart CA, Davis-Street J, Wolfe RR (1996). Prolonged bed rest decreases skeletal muscle and whole body protein synthesis. Am J Physiol.

[8] Ali NA, O’Brien JM, Hoffmann SP, Phillips G, Garland A, Finley JCW (2008). Acquired weakness, handgrip strength, and mortality in critically ill patients. Am J Respir Crit Care Med.

[9] De Jonghe B, Bastuji-Garin S, Sharshar T, Outin H, Brochard L (2004). Does ICU-acquired paresis lengthen weaning from mechanical ventilation?. Intensive Care Med.

[10] Hermans G, Van Mechelen H, Clerckx B, Vanhullebusch T, Mesotten D, Wilmer A (2014). Acute outcomes and 1-year mortality of intensive care unit–acquired weakness: a cohort study and propensity-matched analysis. Am J Respir Crit Care Med.

[11] Burtin C, Clerckx B, Robbeets C, Ferdinande P, Langer D, Troosters T (2009). Early exercise in critically ill patients enhances short-term functional recovery*. Crit Care Med.

[12] Morris PE, Goad A, Thompson C, Taylor K, Harry B, Passmore L (2008). Early intensive care unit mobility therapy in the treatment of acute respiratory failure*. Crit Care Med.

[13] Petersen AMW, Pedersen BK (2005). The anti-inflammatory effect of exercise. J Appl Physiol.

[14] Schweickert WD, Pohlman MC, Pohlman AS, Nigos C, Pawlik AJ, Esbrook CL (2009). Early physical and occupational therapy in mechanically ventilated, critically ill patients: a randomised controlled trial. Lancet.

[15] Moss M, Nordon-Craft A, Malone D, Van Pelt D, Frankel SK, Warner ML (2016). A randomized trial of an intensive physical therapy program for patients with acute respiratory failure. Am J Respir Crit Care Med.

[16] Romer LM, Polkey MI (2008). Exercise-induced respiratory muscle fatigue: implications for performance. J Appl Physiol.

[17] Thille AW, Rodriguez P, Cabello B, Lellouche F, Brochard L (2006). Patient-ventilator asynchrony during assisted mechanical ventilation. Intensive Care Med.

[18] Sinderby C, Navalesi P, Beck J, Skrobik Y, Comtois N, Friberg S (1999). Neural control of mechanical ventilation in respiratory failure. Nat Med.

[19] Terzi N, Pelieu I, Guittet L, Ramakers M, Seguin A, Daubin C (2010). Neurally adjusted ventilatory assist in patients recovering spontaneous breathing after acute respiratory distress syndrome: physiological evaluation*. Crit Care Med.

[20] Spahija J, de Marchie M, Albert M, Bellemare P, Delisle S, Beck J (2010). Patient-ventilator interaction during pressure support ventilation and neurally adjusted ventilatory assist*. Crit Care Med.

[21] Xirouchaki N, Kondili E, Vaporidi K, Xirouchakis G, Klimathianaki M, Gavriilidis G (2008). Proportional assist ventilation with load-adjustable gain factors in critically ill patients: comparison with pressure support. Intensive Care Med.

[22] Costa R, Spinazzola G, Cipriani F, Ferrone G, Festa O, Arcangeli A (2011). A physiologic comparison of proportional assist ventilation with load-adjustable gain factors (PAV+) versus pressure support ventilation (PSV). Intensive Care Med.

[23] Kondili E, Prinianakis G, Alexopoulou C, Vakouti E, Klimathianaki M, Georgopoulos D (2006). Respiratory load compensation during mechanical ventilation–proportional assist ventilation with load-adjustable gain factors versus pressure support. Intensive Care Med.

[24] Hawkins P, Johnson LC, Nikoletou D, Hamnegård C-H, Sherwood R, Polkey MI (2002). Proportional assist ventilation as an aid to exercise training in severe chronic obstructive pulmonary disease. Thorax.

[25] Moderno EV, Yamaguti WPS, Schettino GPP, Kairalla RA, Martins MA, Carvalho CRR (2010). Effects of proportional assisted ventilation on exercise performance in idiopathic pulmonary fibrosis patients. Respir Med.

[26] Dreher M, Kabitz H-J, Burgardt V, Walterspacher S, Windisch W (2010). Proportional assist ventilation improves exercise capacity in patients with obesity. Respiration.

[27] Bianchi L, Foglio K, Pagani M, Vitacca M, Rossi A, Ambrosino N (1998). Effects of proportional assist ventilation on exercise tolerance in COPD patients with chronic hypercapnia. Eur Respir J.

[28] Younes M, Tobin MJ (2013). Proportional-assist ventilation. Principles and practice of mechanical ventilation.

[29] Ingle L (2007). Theoretical rationale and practical recommendations for cardiopulmonary exercise testing in patients with chronic heart failure. Heart Fail Rev.

[30] Herridge MS, Tansey CM, Matté A, Tomlinson G, Diaz-Granados N, Cooper A (2011). Functional disability 5 years after acute respiratory distress syndrome. N Engl J Med.

[31] Gomez-Cabrera M-C, Domenech E, Viña J (2008). Moderate exercise is an antioxidant: upregulation of antioxidant genes by training. Free Radic Biol Med.

[32] McWilliams D, Weblin J, Atkins G, Bion J, Williams J, Elliott C (2015). Enhancing rehabilitation of mechanically ventilated patients in the intensive care unit: a quality improvement project. J Crit Care.

[33] Chiang L-L, Wang L-Y, Wu C-P, Wu H-D, Wu Y-T (2006). Effects of physical training on functional status in patients with prolonged mechanical ventilation. Phys Ther.

[34] Denehy L, Skinner EH, Edbrooke L, Haines K, Warrillow S, Hawthorne G (2013). Exercise rehabilitation for patients with critical illness: a randomized controlled trial with 12 months of follow-up. Crit Care.

[35] Morris PE, Griffin L, Berry M, Thompson C, Hite RD, Winkelman C (2011). Receiving early mobility during an intensive care unit admission is a predictor of improved outcomes in acute respiratory failure. Am J Med Sci.

[36] Gosselink R, Bott J, Johnson M, Dean E, Nava S, Norrenberg M (2008). Physiotherapy for adult patients with critical illness: recommendations of the European Respiratory Society and European Society of Intensive Care Medicine Task Force on Physiotherapy for Critically Ill Patients. Intensive Care Med.

[37] Needham DM, Wang W, Desai SV, Mendez-Tellez PA, Dennison CR, Sevransky J (2007). Intensive care unit exposures for long-term outcomes research: development and description of exposures for 150 patients with acute lung injury. J Crit Care.

[38] on behalf of the Australia and Scotland ICU Physiotherapy Collaboration, Harrold ME, Salisbury LG, Webb SA, Allison GT. Early mobilisation in intensive care units in Australia and Scotland: a prospective, observational cohort study examining mobilisation practises and barriers. Crit Care. 2015 Dec [cited 2015 Dec 18];19(1). http://ccforum.com/content/19/1/336. 10.1186/s13054-015-1033-3 PMC4570617 26370550

[39] Winkelman C, Johnson KD, Hejal R, Gordon NH, Rowbottom J, Daly J (2012). Examining the positive effects of exercise in intubated adults in ICU: a prospective repeated measures clinical study. Intensive Crit Care Nurs.

[40] Camargo Pires-Neto R, Fogaça Kawaguchi YM, Sayuri Hirota A, Fu C, Tanaka C, Caruso P (2013). Very early passive cycling exercise in mechanically ventilated critically ill patients: physiological and safety aspects—a case series. Lucia A, editor. PLoS One.

[41] Harms CA, Wetter TJ, St Croix CM, Pegelow DF, Dempsey JA (2000). Effects of respiratory muscle work on exercise performance. J Appl Physiol.

[42] Harms CA, Babcock MA, McClaran SR, Pegelow DF, Nickele GA, Nelson WB (1997). Respiratory muscle work compromises leg blood flow during maximal exercise. J Appl Physiol.

[43] Romer LM, Lovering AT, Haverkamp HC, Pegelow DF, Dempsey JA (2006). Effect of inspiratory muscle work on peripheral fatigue of locomotor muscles in healthy humans. J Physiol.

[44] Wetter TJ, Harms CA, Nelson WB, Pegelow DF, Dempsey JA (1999). Influence of respiratory muscle work on VO(2) and leg blood flow during submaximal exercise. J Appl Physiol.

[45] Ciavaglia CE, Guenette JA, Langer D, Webb KA, Alberto Neder J, O’Donnell DE (2014). Differences in respiratory muscle activity during cycling and walking do not influence dyspnea perception in obese patients with COPD. J Appl Physiol.

[46] Parshall MB, Schwartzstein RM, Adams L, Banzett RB, Manning HL, Bourbeau J (2012). An Official American Thoracic Society Statement: Update on the Mechanisms, Assessment, and Management of Dyspnea. Am J Respir Crit Care Med.

[47] Blanch L, Villagra A, Sales B, Montanya J, Lucangelo U, Luján M (2015). Asynchronies during mechanical ventilation are associated with mortality. Intensive Care Med.

[48] Vaporidi K, Babalis D, Chytas A, Lilitsis E, Kondili E, Amargianitakis V (2017). Clusters of ineffective efforts during mechanical ventilation: impact on outcome. Intensive Care Med.

[49] Thille AW, Cabello B, Galia F, Lyazidi A, Brochard L (2008). Reduction of patient-ventilator asynchrony by reducing tidal volume during pressure-support ventilation. Intensive Care Med.

[50] Wrigge H, Golisch W, Zinserling J, Sydow M, Almeling G, Burchardi H (1999). Proportional assist versus pressure support ventilation: effects on breathing pattern and respiratory work of patients with chronic obstructive pulmonary disease. Intensive Care Med.

[51] Varelmann D, Wrigge H, Zinserling J, Muders T, Hering R, Putensen C (2005). Proportional assist versus pressure support ventilation in patients with acute respiratory failure: cardiorespiratory responses to artificially increased ventilatory demand. Crit Care Med.

[52] Carteaux G, Córdoba-Izquierdo A, Lyazidi A, Heunks L, Thille AW, Brochard L (2016). Comparison between neurally adjusted ventilatory assist and pressure support ventilation levels in terms of respiratory effort. Crit Care Med.

[53] Beloncle F, Akoumianaki E, Rittayamai N, Lyazidi A, Brochard L (2016). Accuracy of delivered airway pressure and work of breathing estimation during proportional assist ventilation: a bench study. Ann Intensive Care.

[54] Kaminsky DA, Knyazhitskiy A, Sadeghi A, Irvin CG (2014). Assessing maximal exercise capacity: peak work or peak oxygen consumption?. Respir Care.

[55] Hansen JE, Casaburi R, Cooper DM, Wasserman K (1988). Oxygen uptake as related to work rate increment during cycle ergometer exercise. Eur J Appl Physiol.

[56] Katz BZ, Boas S, Shiraishi Y, Mears CJ, Taylor R (2010). Exercise tolerance testing in a prospective cohort of adolescents with chronic fatigue syndrome and recovered controls following infectious mononucleosis. J Pediatr.

[57] Jones NL, Killian KJ (2000). Exercise limitation in health and disease. N Engl J Med.

[58] Torkington M, MacRae M, Isles C (2006). Uptake of and adherence to exercise during hospital haemodialysis. Physiotherapy.

