# Supplementary material for: Can proportional ventilation modes facilitate exercise in critically ill patients? A physiological cross-over study: Pressure support versus proportional ventilation during lower limb exercise in ventilated critically ill patients
Source: Ann Intensive Care. 2017 Jun 12;7:64. doi: 10.1186/s13613-017-0289-y (PMC5468357; doi:10.1186/s13613-017-0289-y)
Supplement: Supplementary file 1 — Additional file 1: Table S1. Results on dyspnea, limb fatigue and distance cycled. Data are presented as median (IQR) values. PROP; Proportional ventilator mode (either Proportional Assist Ventilation or Neurally Adjusted Ventilator Assist). PSV; Pressure Support Ventilation. [file 13613_2017_289_MOESM1_ESM.docx]

Supplemental Digital Content

**Can Proportional Ventilation Modes Facilitate Exercise in Critically Ill Patients? A Physiological cross-over study.**

Pressure Support versus Proportional Ventilation during lower limb exercise in ventilated critically ill patients.

**E. AKOUMIANAKI^1^, N. DOUSSE, A. LYAZIDI^2^, JC LEFEBVRE^1^, S. GRAF ^1^, R.L. CORDIOLI^1^, N. REY^1^, JCM. RICHARD^2^, L. BROCHARD^2^**

METHODS

*Patients*

The study took place at the Geneva university hospital ICU, a mixed medico-surgical ICU. Over an 18-month period, critically ill patients requiring assisted MV for more than 48 hrs, who were anticipated to continue receiving MV for the next 24hours and were submitted to re-entrainment by physical therapists were prospectively enrolled. As part of routine practice, all mechanically ventilated patients were submitted to rehabilitation training with cycloergometer provided that there were no contraindications to exercise (severe hypoxaemia, respiratory distress, rescue therapies for respiratory insufficiency such as NO, high frequency oscillatory ventilation, prone positioning or ECMO, cardiac or neurological contraindications to exercise, conditions impairing cycling movement, active bleeding). A detailed description of inclusion and exclusion criteria is provided below.

**Inclusion criteria**

- Male or female patient.
- Age: 18-85 years.
- Patient admitted to the intensive care unit (ICU).
- Spontaneously breathing patient, under partial ventilatory support and mechanically ventilated through an entotracheal tube or a tracheostomy.
- Patient with stable hemodynamics.
- Patient intubated and mechanically ventilated for ≥ 48hrs at the time of randomization and anticipated to continue receiving MV for the next 24 hours.
- Collaborative patient with adequate neurologic status.
- Written informed consent signed and dated by the patient will be requested before patient inclusion when a full comprehension of the investigator explanations was possible. When signature was not possible, consent taken orally or by gesture requested first and formal written consent requested as soon as the patient was able to provide written informed consent.
- Written informed consent signed and dated by the patient next of kin after full explanation of the study by the investigator and prior to study participation was mandatory for every patient.
- Written informed consent signed and dated by the attending physician, certifying his agreement to the participation of the patient to this study.

**Exclusion criteria**

- Hemodynamic instability:
  - > 20% variation of mean arterial pressure and/or heart rate (HR) in the last 2 h
  - HR at rest < 50 or > 120 beats per minute (bpm)
  - Vasoactive or inotropic agent use
- Usual cardiac contraindication to exercise:
  - Malignant arrhythmia in the preceding 24 hours
  - Symptoms, positive troponins or ECG abnormalities suggestive of active myocardial ischemia
  - Severe aortic stenosis or severe hypertrophic obstructive cardiomyopathy
  - Dissecting aortic aneurysm or recent aortic surgery
  - Acute or suspected myocarditis or pericarditis
  - Acute uncontrolled heart failure
- Positive end-expiratory pressure (PEEP) ≥ 8 cmH_2_O.
- SaO_2_ < 95% with FiO_2_ ≥ 50%.
- Symptoms of respiratory distress or respiratory rate ≥ 30 breaths/min while on adequate ventilatory support.
- Active uncontrolled systemic infection.
- Conditions impairing the cycling movement:
  - Trauma or surgery of leg, pelvis or spine
  - Body length < 1.5 meter
  - Open abdominal wounds
  - Significant wounds on lower limbs or sacrum
  - Body mass index (BMI) > 35
  - Femoral central venous or arterial catheter
- Proximal lower limb deep vein thrombosis.
- Intracranial hypertension.
- Presence of a known esophageal problem, active upper gastrointestinal bleeding or any other contraindication to the insertion of a nasogastric tube.

**Study protocol**

At day 1 the patients underwent an incremental workload test on a cycloergometer (MOTOmed letto 2) to determine the maximum resistance level capacity. This started from passive mobilization on the bicycle (no resistance) and the resistance was gradually increased based on patient's tolerance. The cycle ergometer has 20 different resistive load steps; loading was increased step-by-step each minute upon completion of the exercise period. Patients were instructed to cycle between 30 to 60 rates per minute (RPM). During this session, patients were ventilated with PSV. Ventilator settings during this session were selected as follows: (a) Flow triggering set as the most sensitive possible not associated without promoting autotriggering (b) Pressure support leading to a tidal volume (VT) of 6-8 ml/kg and a respiratory rate lower than 30 breaths/min and (c) cycling-off criterion of 25% (40% for patients with Chronic Obstructive Pulmonary Disease).

The day after, 2 exercise sessions of 15 minutes (min) at a constant workload were conducted in a random order, each separated from the other by a 60 min resting period. The workload imposed represented 60% of the maximal workload attained on the previous day on the incremental test, to ensure staying away from a risk of fatigue. During each exercise session patients were ventilated with PSV and a proportional mode in random order. The type of proportional mode (PAV+ or NAVA) was randomly selected. To ensure patient safety, the test could be stopped before the end of any 15 min session, based on well-defined stopping criteria, notably patient perception of a severe dyspnea or discomfort (see below). Oesophageal NAVA catheter was inserted and positioned for patients randomized to NAVA. A 60 min resting period elapsed between the two exercise periods. Forty minutes after the end of the first exercise session, the ventilator changed to the other mode, allowing a 20 min adaptation period at rest before starting the second exercise session. The physiotherapist supervising the exercise test was blinded to the attributed sequence and the ventilator screen was hidden so that it was not possible for him to know the ventilation mode tested. The patient was not informed of the mode. Only the investigator in charge of collecting the data was aware of the mode.

Ventilator settings with PSV were the same as in day 1. During ventilation with PAV+, flow triggering was similar to that during PSV, the support was titrated to attain the same mean airway pressure (Paw_mean_) as in PSV and and cycling off was set at 3lt/min. With NAVA, flow triggering threshold was similar to PSV, EAdi triggering was 0.5 µV while cycling off was fixed at 70% of peak EAdi. In all ventilator modes PEEP remained at the same level already described by the attending physician and FiO_2_ was titrated to target SpO_2_ > 95% before starting the exercise test (FiO_2_ ≤ 50%).

Standardized verbal encouragements were provided by the physiotherapist every 2 min.

**Criteria to stop the exercise session**

The exercise period could be stopped prematurely as soon as one of these conditions was met:

- Patient made a sign (thumb down) indicating he wants to stop exercising.
- Patient stopped pedaling or the cycling rate fell below 30RPM for 20 consecutive seconds despite standardized verbal encouragements.
- Abnormal physiologic response:

Heart rate (HR) > 140 bpm

>20% decrease in HR

Systolic blood pressure (SBP) > 200 mmHg

>20% decrease in systolic or diastolic blood pressure

Significant arrhythmia

Symptoms of myocardial ischemia, such as new chest pain

Respiratory distress on the ventilator (RR > 40 / min, sustained use of accessory respiratory muscles, otherwise apparent respiratory distress)

SpO_2_ <90% for ≥10s

- At any time the physiotherapist and/or the attending physician judged that pursuing exercise exposed patient to unacceptable risk.

**Equipment**

All exercise tests were made using the same bedside cycle ergometer (MOTOmed Letto 2, RECK-Technik GmbH and Co. Betzenweiler, Germany). An integrated on board computer displayed all the relevant data concerning the exercise session. This cycle ergometer is specifically designed to be used in bedridden patients and rests firmly on the ground at the foot of the bed to ensure stability. Calves were properly placed to stabilize the leg movement. The MOTOmed Letto 2 allows the setting of 20 different resistive levels to increase the workload. An integrated onboard computer displays all the relevant data concerning the exercise session, such as exercise time and power generated (watts). These data were manually collected during the session.

Patients randomized to PSV/PAV+ were ventilated with a PB 840 ventilator (Covidien, Mansfield, MA, USA). Patients randomized to PSV/NAVA were ventilated with a Servo-i ventilator (Maquet, Solna, Sweden).

An indirect calorimetry apparatus was used to measure oxygen uptake (Quark RMR ICU; Cosmed, Rome, Italy). The calorimeter measured the oxygen consumption by determining the changes in volume within a closed system. Before obtaining the measurements the calorimeter was calibrated, as specified by the manufacturer.VO_2_ was determined by the difference between the quantity of oxygen flowing into the patient (FiO_2_) and the quantity flowing out (FeO_2_); VO_2_= [Volume_in_ • FiO_2_] - [Volume_out_ • FeO_2_], where FiO2 and FeO2 are the fractional concentration of O2 in inspired and expired gas, respectively. Before obtaining the measurements the calorimeter was calibrated, as specified by the manufacturer.

Airway flow and pressure sensors were connected to the respiratory circuit, proximal to the Y-piece. The flow was measured through a pneumotachograph (Fleish No. 2; Metabo; Epalinges, Switzerland). Proximal airway pressure were measured using a differential pressure transducer (Validyne MP45 ±80cmH_2_O; Northridge, CA, USA). Signals were acquired with an analogue-digital converter (MP100; Biopac systems, Goleta, CA, USA), sampled at 200 Hz and stored in a laptop computer for subsequent off-line analysis (Acqknowledge 3.7.3, Biopac Systems).

Data collection

Exercise performance expressed as mean and peak power generated (watts), distance performed and exercise time (min) were manually collected from the cycle ergometer. Dyspnea and limb fatigue were evaluated through a modified Borg dyspnea scale score and a Borg limb discomfort scale.

Respiratory [respiratory rate (RR) and SpO_2_)] and hemodynamic parameters [heart rate (HR), systolic (SBD), diastolic (DBD) and mean arterial blood pressure (meanBP) and double product (SBD*HR)] were continuously monitored throughout exercise.

Oxygen consumption (VO_2_) and CO_2_ production (VCO_2_) were measured through indirect calorimetry. The % change in VO_2_ (∆VO_2_%) between baseline and the final 5 minutes of each exercise session and the work efficiency (ratio of ∆VO_2_ per power generated) were computed.

Breath-by-breath analysis was performed on the recorded data by using Acqknowledge® software (Biopac Systems Inc., Goleta, CA, USA). The following variables were determined from the flow signal: tidal volume (V_T_), mechanical inspiratory (Ti), duty cycle (Ti/Ttot) and minute ventilation (VE). Peak (Ppeak) and mean airway pressure (Pmean) during inspiration were computed from the Paw signal.

*Asynchrony*

To assess the severity of asynchrony we used the asynchrony index (AI) computed by the ratio of the total number of all asynchrony events (ineffective efforts, double triggering, autotriggering, short and prolonged cycles) by the total number of ventilator cycles (triggered or not), expressed as a percentage.

Asynchrony detection was made visually, based on Flow and Paw signals. The point at which inspiratory flow became positive represented the onset of mechanical inspiration while the transition from positive to negative flow the end of mechanical inspiration. Inspiratory efforts that failed to trigger the ventilator were termed as ineffective efforts (IE). Double-triggering was defined as two pneumatic cycles separated by a very short expiratory time (defined as less than one-half of the mean inspiratory time) triggered by one patient effort. Autotriggered cycles were those delivered by the ventilator without a prior decrease in Paw signal. Short and prolonged cycles were defined as cycles with Ti_mech_ less than one-half or greater than twice the mean Ti_mech_, respectively. Asynchrony events were evaluated over the entire 15min exercise session.

**SUPPLEMENTARY RESULTS**

The perception of dyspnoea at baseline (median, IQR) did not differ between Proportional (3.5, 1.3-6.0) and PSV (4.5, 2.3-5.0) ventilation. There was also no statistically significant difference on the perception of dyspnoea during exercise with Proportional (5.0, 4.3-6.0) and PSV ventilation (5.0, 5.0-6.0). Similarly, baseline perception of limb fatigue between Proportional (5.0, 0.5-5.8) and PSV ventilation (5.0, 0.3-7.0) as well as perception of limb fatigue during exercise with Proportional (4.0, 0.3-7.0) and PSV ventilation (5.5, 2.3-7.5) were the same.

Table 1 Supplement. Results on dyspnea, limb fatigue and distance cycled.

| **VARIABLE TESTED** | **Proportional PSV** | | ***p*** |
| --- | --- | --- | --- |
| Distance (km) | 3.3 (2.3-3.6) | 3.3 (2.6 -3.9) | 0.09 |
| Dyspnea | 1 (-0.8-3.8) | 2 (0.5-4) | 0.33 |
| Limb fatigue | 0.5 (0-1.8) | 2 (0.3-2) | 0.53 |

Data are presented as median (IQR) values. PROP; Proportional ventilator mode (either Proportional Assist Ventilation or Neurally Adjusted Ventilator Assist). PSV; Pressure Support Ventilation.
